# 3D geometric modelling and clinical validation of lower limb and preliminary 2‐year post‐operative clinical outcomes of neutral boundary alignment in total knee arthroplasty

**DOI:** 10.1002/jeo2.70652

**Published:** 2026-02-27

**Authors:** Bruno Violante, Lorenzo Deveza, Francesco Pollara, Giovanni Rusconi, Gianroberto Ferreri, Alessandro Annibaldi, Ilwhan Park

**Affiliations:** ^1^ ESSKA‐EKA Board Chair; ^2^ Orthopedic Department Hospital Isola Tiberina ‐ Gemelli Isola – Center for Knee and Hip Reconstructive Surgery and Traumatology Roma Italy; ^3^ Department of Orthopaedic Surgery Baylor College of Medicine Texas USA; ^4^ Radiology Department Pineta Grande Hospital Via Vecchia S. Gennaro Pozzuoli Italy; ^5^ Radiology Department Hospital Isola Tiberina ‐ Gemelli Isola Rome Italy; ^6^ Lento Medical Innovation, Inc Houston Texas USA

**Keywords:** functional alignment, knee alignment, neutral boundary alignment, total knee arthroplasty

## Abstract

**Purpose:**

To introduce a novel three‐dimensional (3D) modelling concept for lower limb alignment in total knee arthroplasty (TKA), termed neutral boundary alignment (NBA) and to present preliminary clinical data supporting this physics‐based custom alignment strategy.

**Methods:**

NBA is defined as the alignment at which the knee joint line is parallel to the ground during the mid‐stance phase of gait, achieving static equilibrium with the NBA axis aligned to gravity. A theoretical geometric model of the lower limb was developed, integrating principles of physics to describe this condition. Preliminary clinical validation was performed using radiographic data from an initial cohort of 12 patients, followed by an additional 45 patients who underwent TKA planned and executed using the NBA concept. Postoperative alignment relative to the ideal parallel joint line, intraoperative need for ligament balancing and clinical outcomes were evaluated. Clinical function was assessed using the forgotten joint score (FJS‐12) at 2 years postoperatively.

**Results:**

Across the combined cohort, postoperative joint line alignment demonstrated an average deviation of 0.1° from the ideal parallel orientation, with a standard deviation of 0.9°. Surgeries performed using NBA consistently achieved stable knees without the need for formal ligament balancing. The mean 2‐year postoperative FJS‐12 was 86 ± 11, indicating a high level of joint ‘forgettability’ in daily activities.

**Conclusions:**

Despite the relatively small sample size, this study supports NBA as a physics‐based, custom alignment method in TKA that closely reproduces a joint line parallel to the ground in mid‐stance, optimises knee stability and preserves soft tissue integrity. These preliminary findings justify further investigation of NBA in larger, prospective cohorts.

**Level of Evidence:**

Level IV.

**Abbreviations**
FA: functional alignment
HKA: hip–knee–ankle
KA: kinematic alignment
MA: mechanical alignment
NBA: neutral boundary alignment
Post‐Op: post‐operative
Pre‐Op: pre‐operative
TKA: total knee arthroplasty
V/V: varus/valgus

## INTRODUCTION

The knee, hip and ankle joints function as passive systems, primarily influenced by potential energy derived from gravity. According to the fundamentals of physics, static equilibrium occurs when forces acting on an object are precisely balanced, allowing the object to remain stable without motion. When a body in a selected inertial frame of reference neither rotates nor moves in translational motion, then the body is in static equilibrium in this frame of reference. Dynamic equilibrium, in contrast, is when an object moves at a constant speed, and all the forces on the object are balanced. The quasi‐static process is referred to as a slow process that happens at an infinitesimally slow rate. Consequently, the quasi‐static assumption can be applied to the static equilibrium analysis under a very slow rate of motion. These are the fundamentals of classical physics which describe the motion of the lower limb.

The success of total knee arthroplasty (TKA) strongly depends on the alignment technique. Over the past few decades, various alignment methods have been proposed, including mechanical alignment (MA) [16], kinematic alignment (KA) [10, 15, 19], functional alignment (FA) [12], anatomical alignment (AA) [3, 4, 11] and so on. Despite these efforts, no clear TKA alignment method has been universally accepted [1].

This study is the extension of the study done by Deveza et al. [5] and introduces neutral boundary alignment (NBA), a novel method grounded in the principles of physics, such as the principle of equilibrium, energy conservation, gait cycle, Ptolemy theorem [8] and so on. In this paper, the authors proposed the sufficient condition (hypothesis), ‘we define the knee stability and balance condition as if the neutral boundary axis is in the direction of gravity and is simultaneously situated within hip, knee and ankle boundaries in the midstance phase of the gait cycle, then the knee is set to be stable and balanced’. NBA is developed to find common ground among MA, KA and AA by being designed to achieve static equilibrium during the mid‐stance phase of the gait cycle, where the joint line is parallel to the ground, and the NBA axis aligns with gravity. Consequently, the aim of NBA is to catch the concepts of MA, KA and AA all in one. This paper presents a theoretical 3‐D model of the lower limb based on NBA and validates NBA through preliminary clinical observations and outcomes. The findings demonstrate that NBA provides a unique, patient‐specific alignment, ensuring knee joint stability and optimal implant positioning without the need for additional ligament balancing procedures. Furthermore, the theoretical model is examined in reference to real‐world and theoretical predictions of NBA, such as ligament MCL and LCL balance (no soft tissue laxity) and the physical joint line [5] being parallel to the ground as an extension of the lower limb, and the clinical data demonstrates the validity of the NBA model.

## METHODS

### Macro property of femur structure

**Figure 1:**
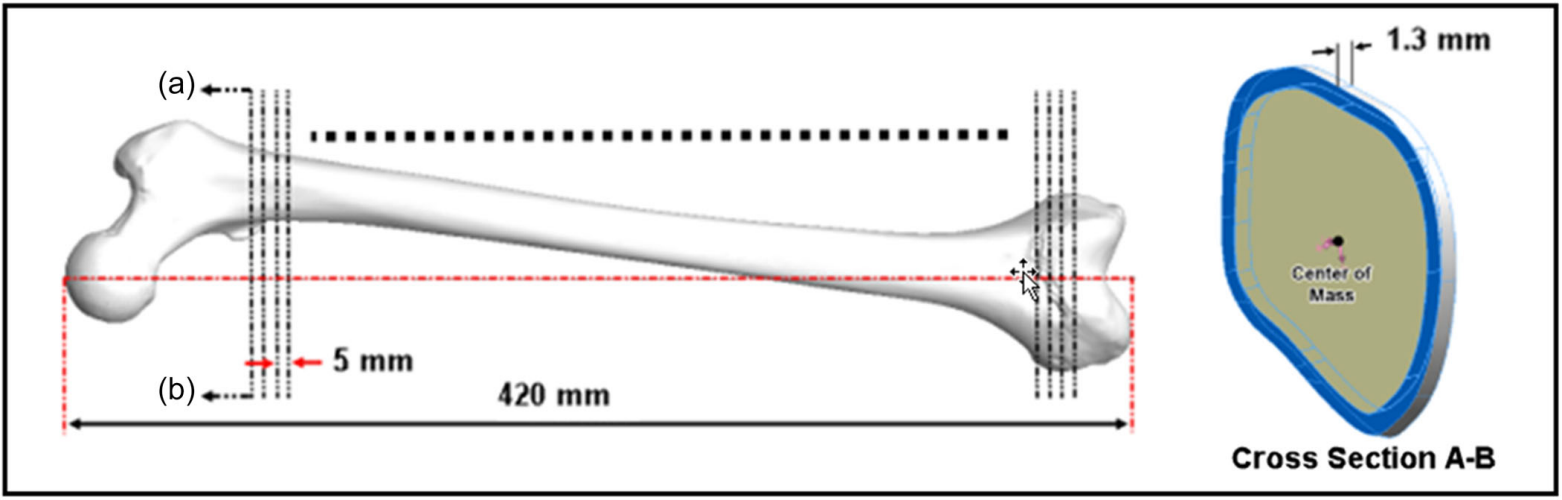
Femur: slices of 5 mm intervals and 1.3 mm thickness at each slice to identify the centre of mass.

The femoral mechanical axis is defined by the line from the centre of the femoral head to the centre of the knee. As shown in Figure 1, the femur model is arranged along the femoral mechanical axis. It follows the femur, sliced perpendicular to the femoral mechanical axis by 5 mm intervals. Each slice, with a thickness of 1.3 mm, is obtained. Under the assumption of homogeneous material properties of cortical and cancellous bone, the centre of mass of each slice is identified using the Mass Properties Tool Option in *Solidworks* CAD software. Each centre of mass is connected using a spline curve, the centre mass curve (CMC) and the characteristics of the CMC are investigated.

**Figure 2:**
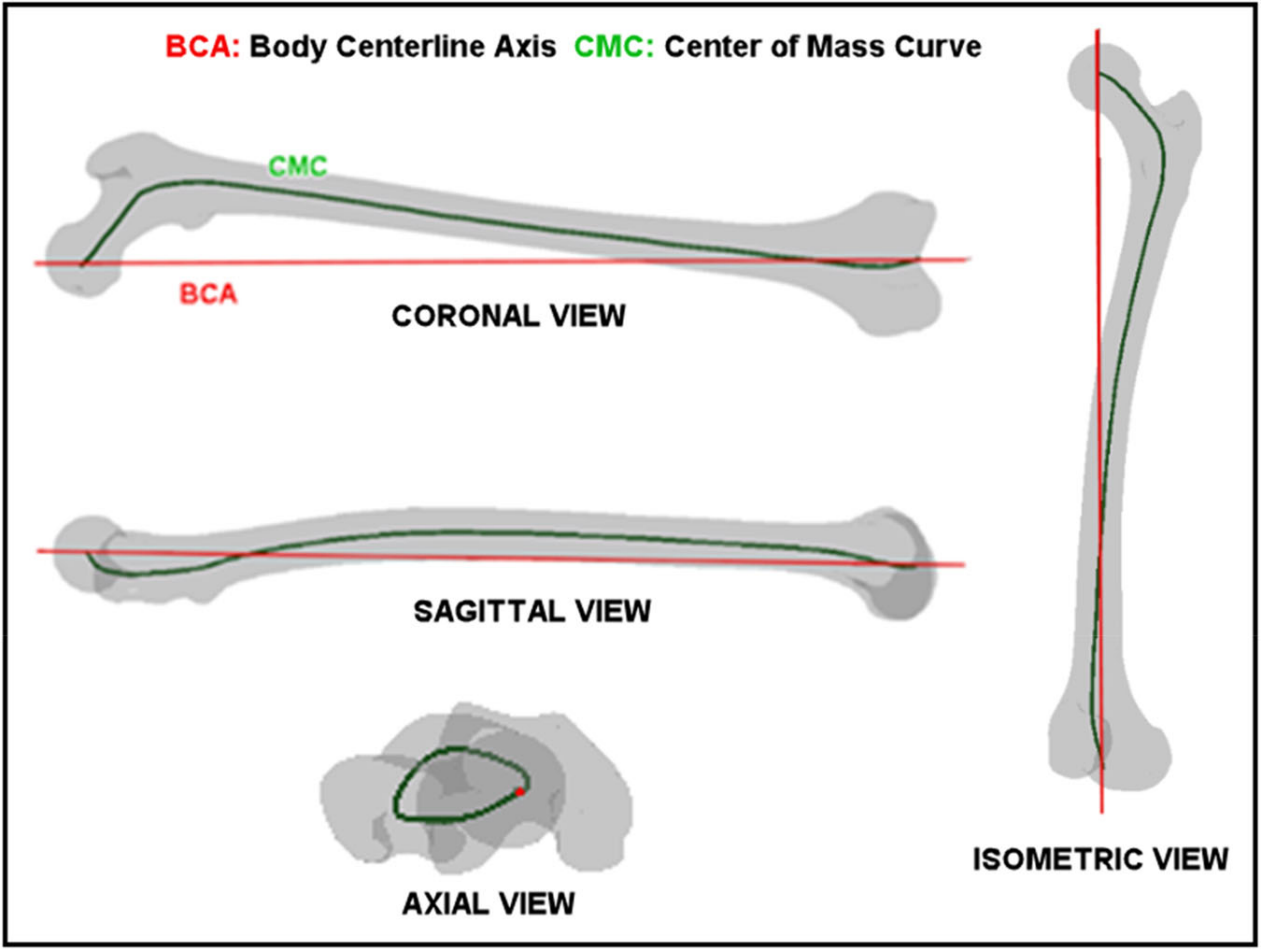
Centre mass curve (CMC) with respect to the body centerline axis (BCA).

Figure 2 displays the coronal, sagittal, axial and isometric views of the CMC inside the femur model. It can be observed that the CMC exhibits a reverse conical shape, completing a 360‐degree rotation akin to a reverse conical spring. The upper femur, including the femoral neck and head, functions similarly to a torsional spring. Overall, the femur would be modelled as a reverse conical torsional spring. This reverse conical configuration, referred to as the body centerline axis (BCA), provides great resistance against transverse shear force and normal compressive force. Furthermore, the femoral microstructure, comprising cortical and cancellous bone, contributes to the resistance against the transverse shear force and normal compressive force [1].

It is important to note that the mechanical property of BCA in conical torsional springs usually represents the direction of the force acting onto the structure. Thus, according to the minimum energy principle and the conservation of energy, it can be deduced that the BCA in Figure 2 should align in the direction of gravity at the mid‐stance of the gait cycle with one leg weight acceptance stage of the lower limb where kinetic energy is stored as potential energy. It should be noted that, as shown in Figure 2, the BCA does not necessarily coincide with femoral mechanical axis, highlighting individual anatomical variations and their biomechanical significance.

### Lower limb modelling at mid‐stance

At the mid‐stance of the gait cycle [20], kinetic energy is temporarily stored as potential energy. To determine the direction of BCA at mid‐stance and establish static equilibrium, a generative structural algorithm along with Ptolemy theorem was applied [8, 9]. The theoretical modelling construction of the NBA is presented by Lorenzo et al. [5, 6]

**Figure 3:**
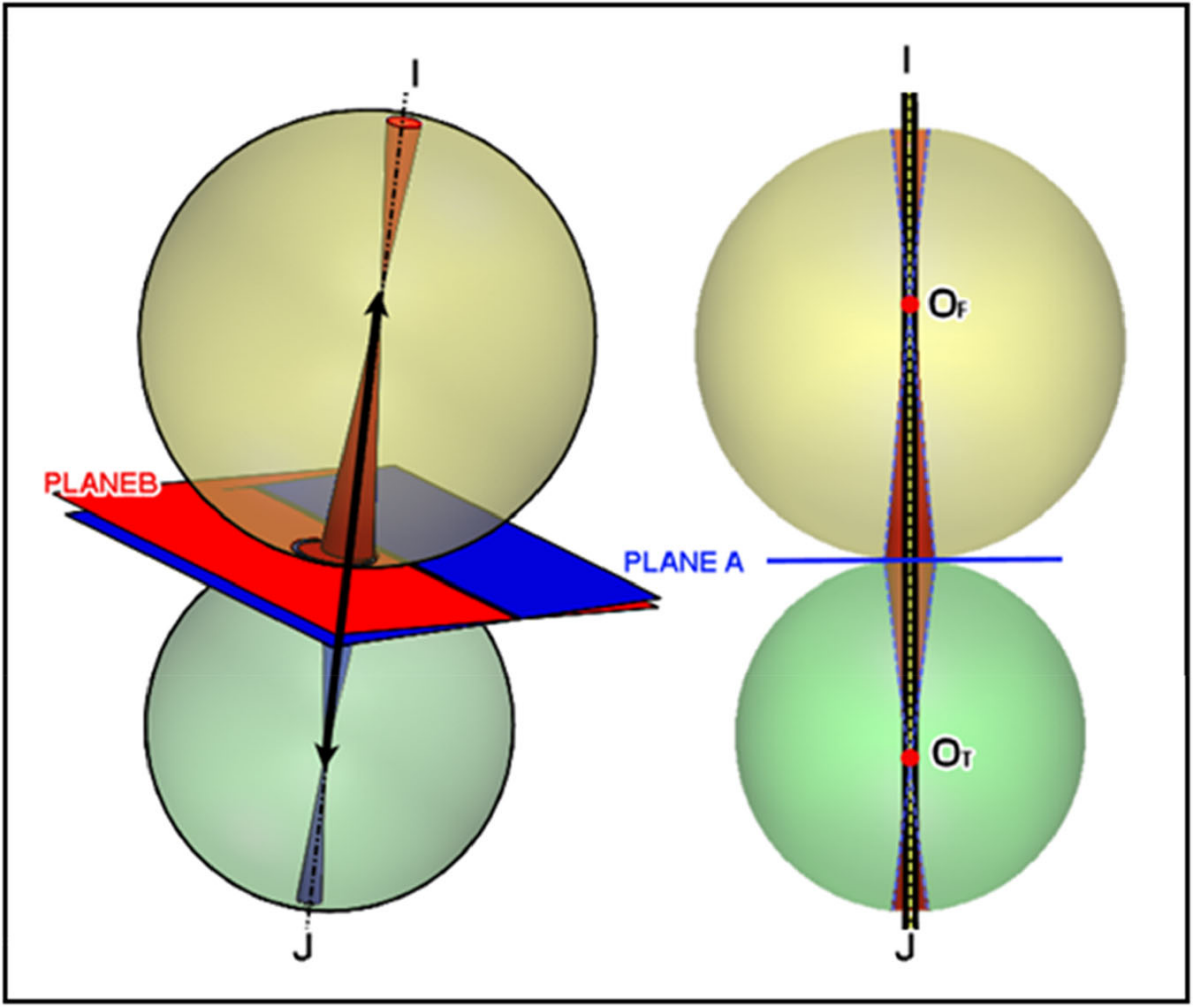
3D representation of the lower limb model at the mid‐stance of the gait cycle.

Furthermore, the two‐dimensional (2‐D) femoral and tibial structures [1] can be elaborated to the three‐dimensional (3‐D) diagram as shown in Figure 3. The 3‐D model shows the femoral and tibial spheres along with the femoral and tibial double cones of point vortices structures. Plane A is defined as the physical joint plane. Figure 3 also exhibits that Plane B represents the tibial slope plane around 3 degrees rotation with respect to Plane A. The validity of the 3‐D model illustrated in Figure 3 is based on Thurston's geometrization conjecture for three‐manifolds [17], famously proven by mathematician Grigori Perelman [14].

According to the mid‐stance of the gait cycle, the knee joint shows no muscle activity, leading to exclusive compression acting on the knee joint while the knee joint exhibits almost zero torque [7]. Hence, the knee joint momentarily is almost at a zero flexion‐extension moment and power and exhibits minimal balanced ground reaction force by the body weight at mid‐stance [18], resulting in the anterior–posterior, adduction–abduction and internal–external rotations being momentarily zero where the MCL and LCL are perfectly balanced in full extension in tension. Furthermore, Figure 3 exhibits the two double cones inside the spheres of the femur and the tibia that are joined together along the NBA axis. Based on the study of the system of point vortices on the hyperboloid [13], the stability of the lower limb 3D model is uniquely defined along the NBA axis. Therefore, under the quasi‐static assumption, the mid‐stance of the gait cycle is the state of static equilibrium at the lower limb achieved by minimising its energy in classical physics according to the minimum energy principle. Hence, the NBA axis at mid‐stance of the gait cycle is in the direction of gravity. The NBA is a patient‐specific alignment since everyone has different contact geometries of the hip, knee and ankle joints although, as shown in Figure 3, Plane A being parallel to the ground is the common factor for everyone.

Over the last 3 years, more than 250 surgeries have been performed using the NBA technique by four surgeons. This study involved the use of *Smith and Nephew Legion* (Symmetric), *Journey 2* (Asymmetric), *Stryker* (Triathlon), *Depuy* (Attune) and *Lepin* (Kneo) TKA implants.

Twelve patients among the 2‐year post‐op patients were randomly selected for detailed radiographic analysis. A total of 12 consecutive radiographs were obtained and evaluated to assess the alignment and validate the NBA model. Weight‐bearing HKA A‐P radiographs were obtained for each patient both pre‐ and post‐operatively (GMM Crisis Evolution radio‐fluoroscopy system). Furthermore, a total of 45 cases among the 2‐year post‐op patients were selected. The patient‐reported outcomes were assessed through the forgotten joint score (FJS‐12).

**Figure 4:**
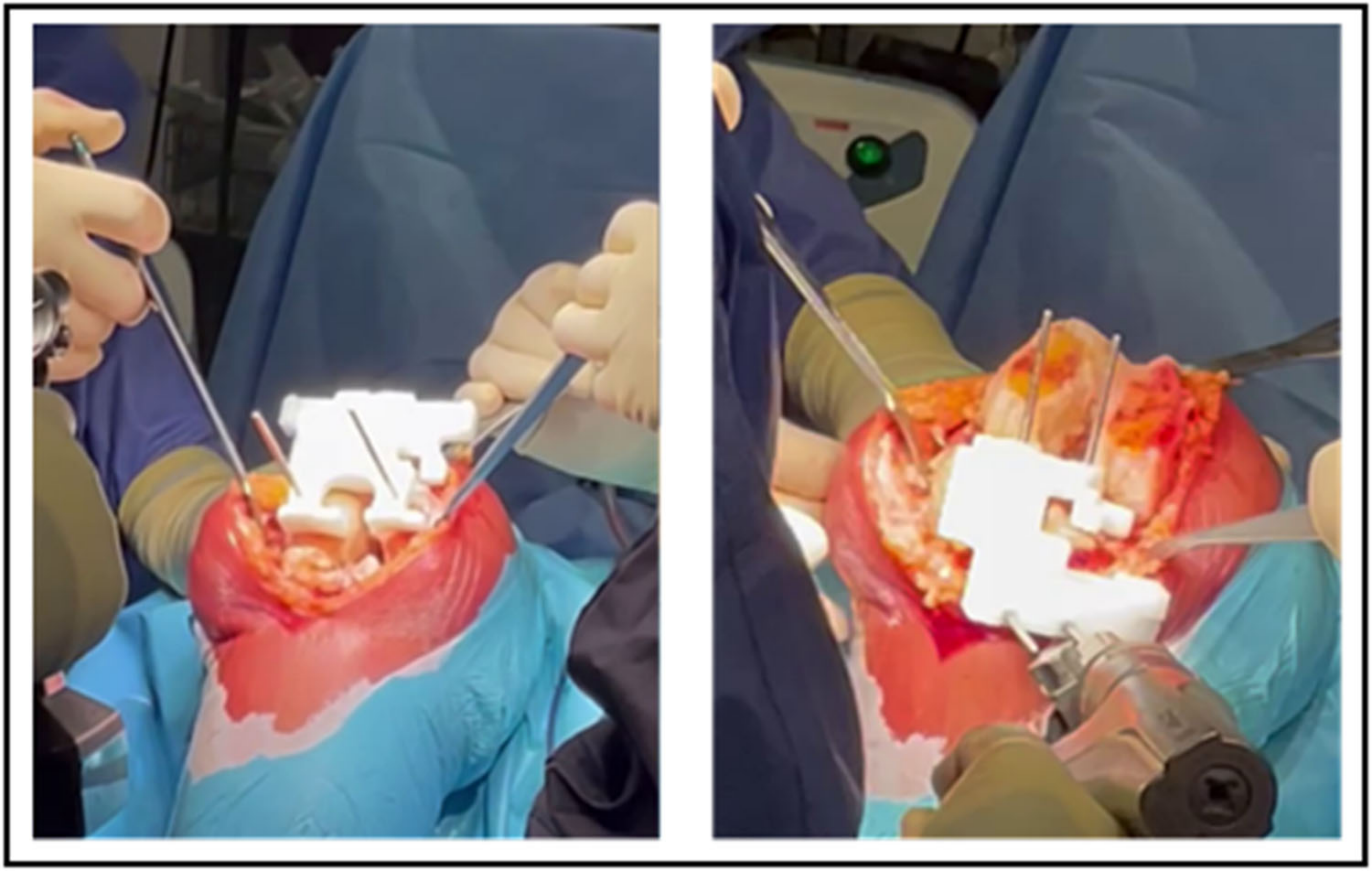
*Ptoleknee* femoral and tibial surgical guides being used in surgery.

In this study, the NBA surgical technique relies on precision tools, *PtoleKnee* cutting guides (*Lento Medical Innovation, Inc*.), to achieve consistent and accurate distal and proximal resections in TKA, as shown in Figure 4. New profiles have been created for each case on the Lento Medical Innovation platform.

## RESULTS

### Radiographic analysis and FJS‐12

**Figure 5:**
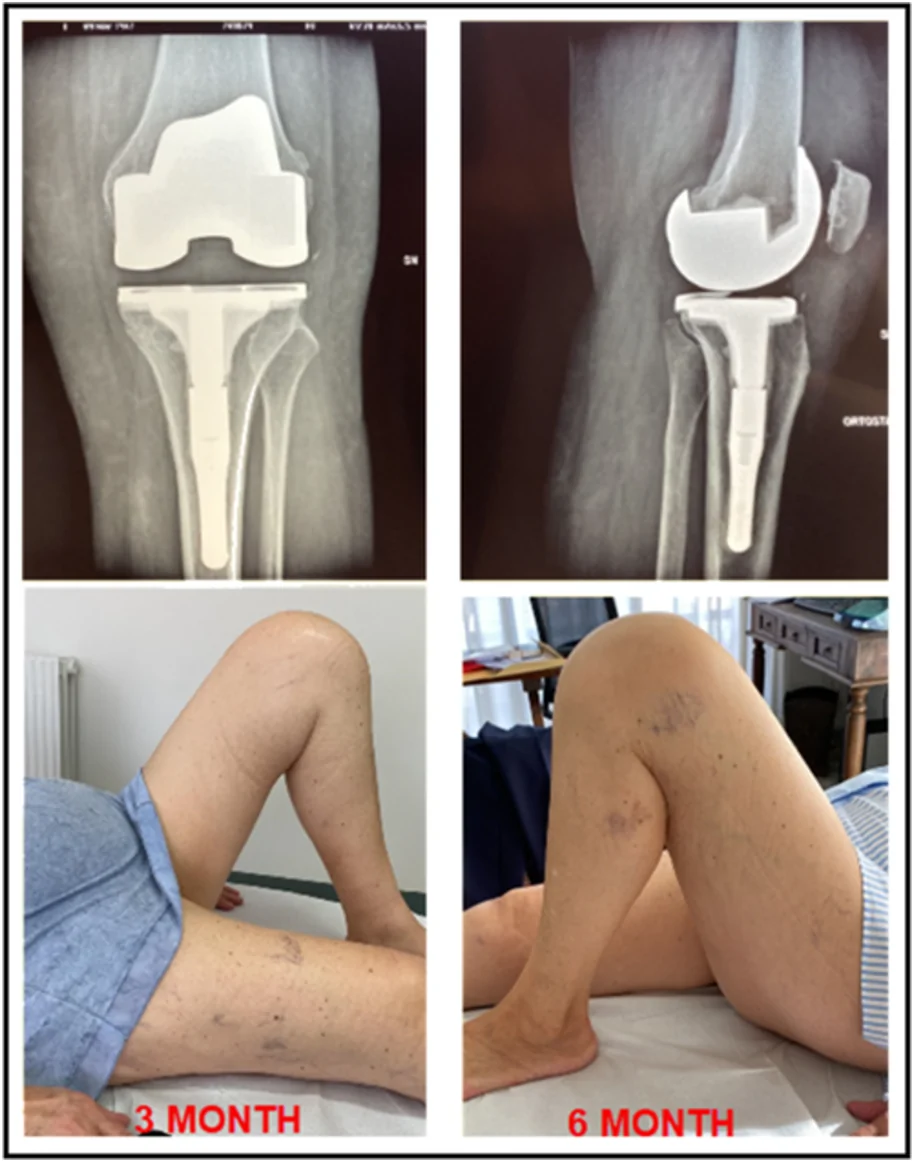
Post‐op radiographs and 3‐ and 6‐month post‐op photographs.

**Figure 6:**
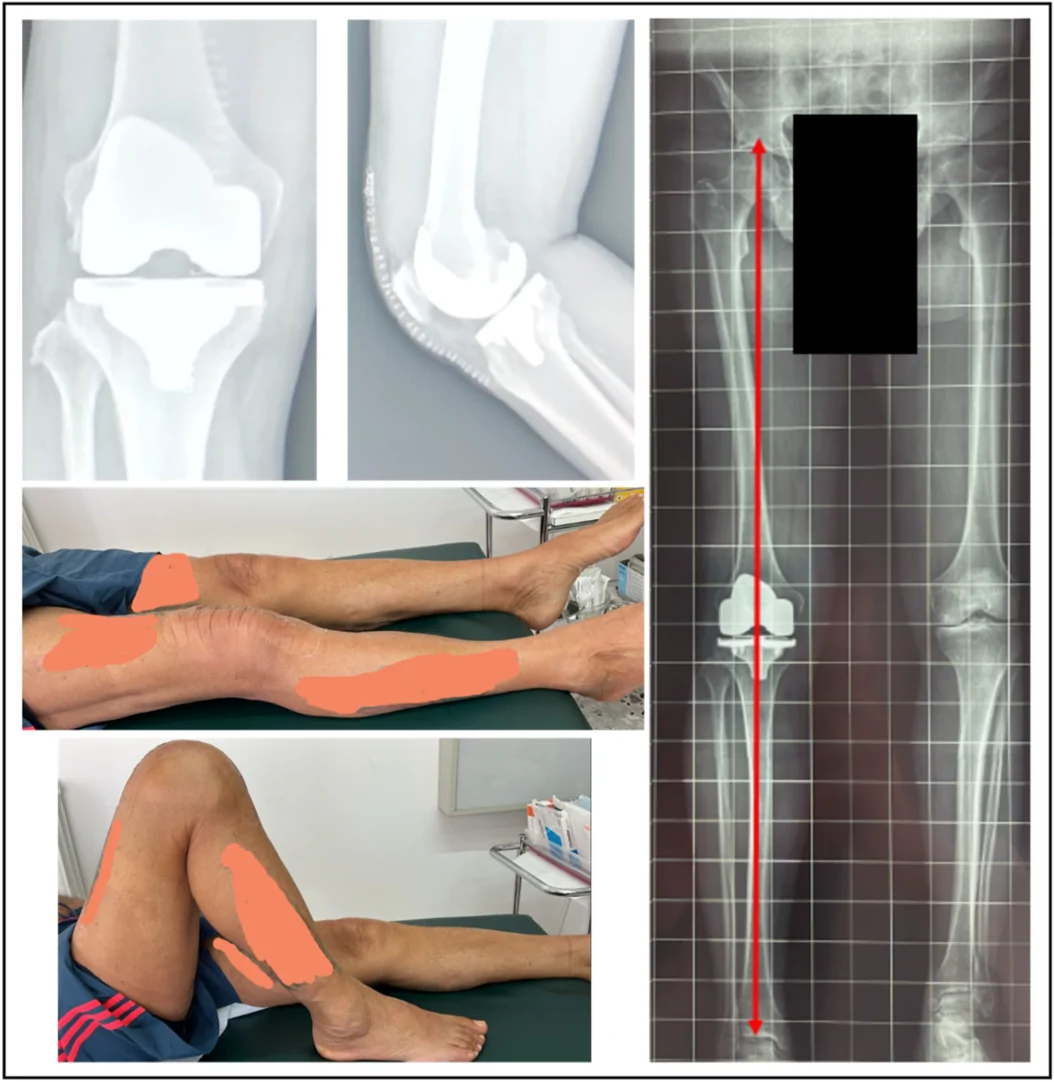
Post‐op radiographs and 3‐month post‐op photographs (*Stryker Triathlon*).

Regardless of the cruciate‐retaining (CR) and posterior‐stabilised (PS) implants, the minimum and maximum flexion angles recorded were 120 and 150 degrees, respectively, with an average of 135 degrees. Notably, no ligament balancing procedures were performed for all the patients, indicating that no tissue laxity was observed under NBA. Figure 5 shows the coronal and sagittal views of radiographs, *Smith and Nephew Journey 2*, and flexion post‐op photographs of 3 and 6 months. It should be noted that CR implants perform better in patella tracking motion than the PS implants. Furthermore, the asymmetric implant (*Smith and Nephew Journey 2*) shows no significant difference compared to the symmetric implant (*Smith and Nephew Legion*) in terms of implant stability. Also Figure 6 shows the coronal and sagittal views of radiographs, *Stryker Triathlon*, and flexion post‐op photographs of 3 months.

**Figure 7:**
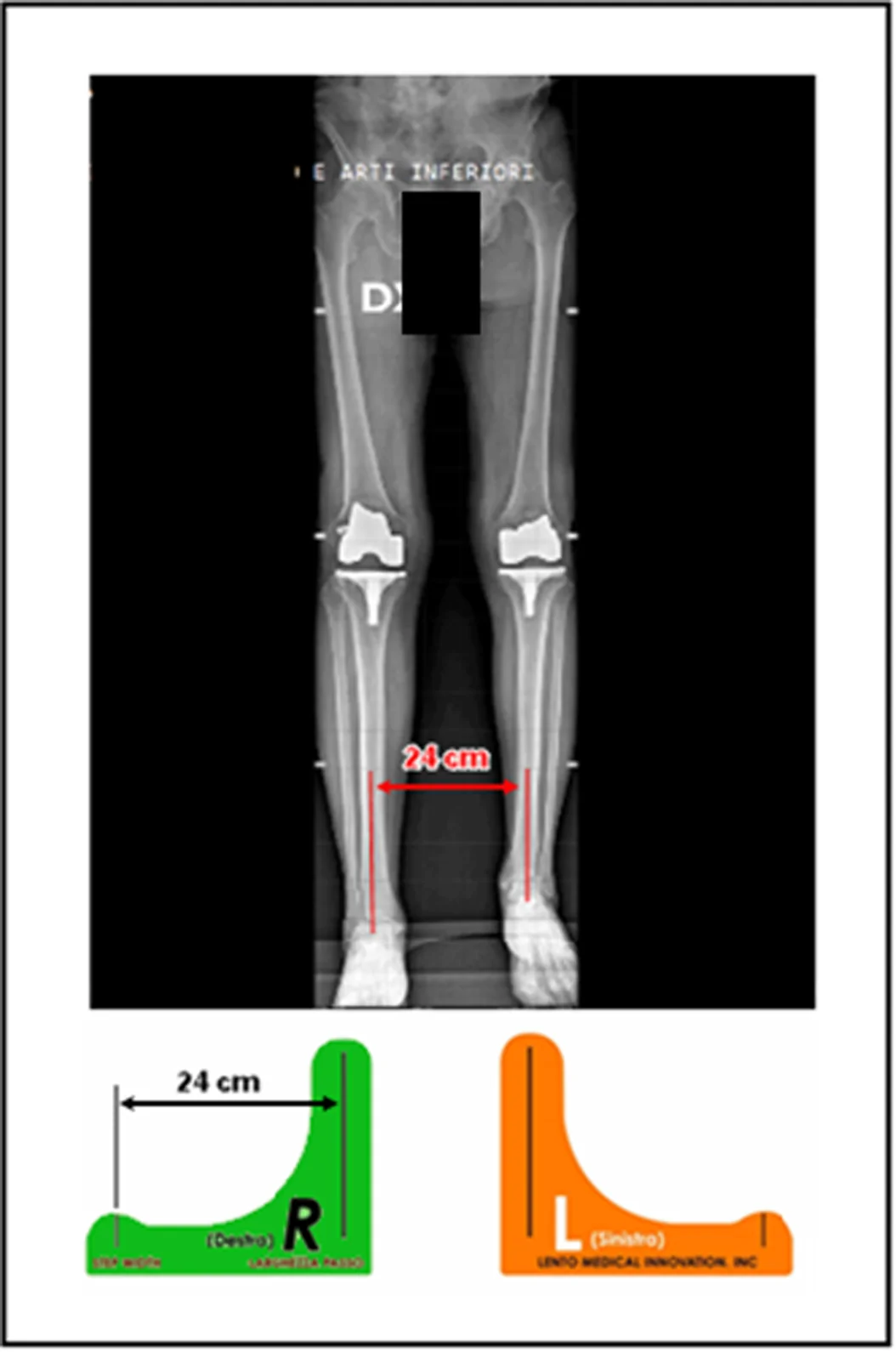
Step width of 24 cm and long leg post‐op radiograph.

**Figure 8:**
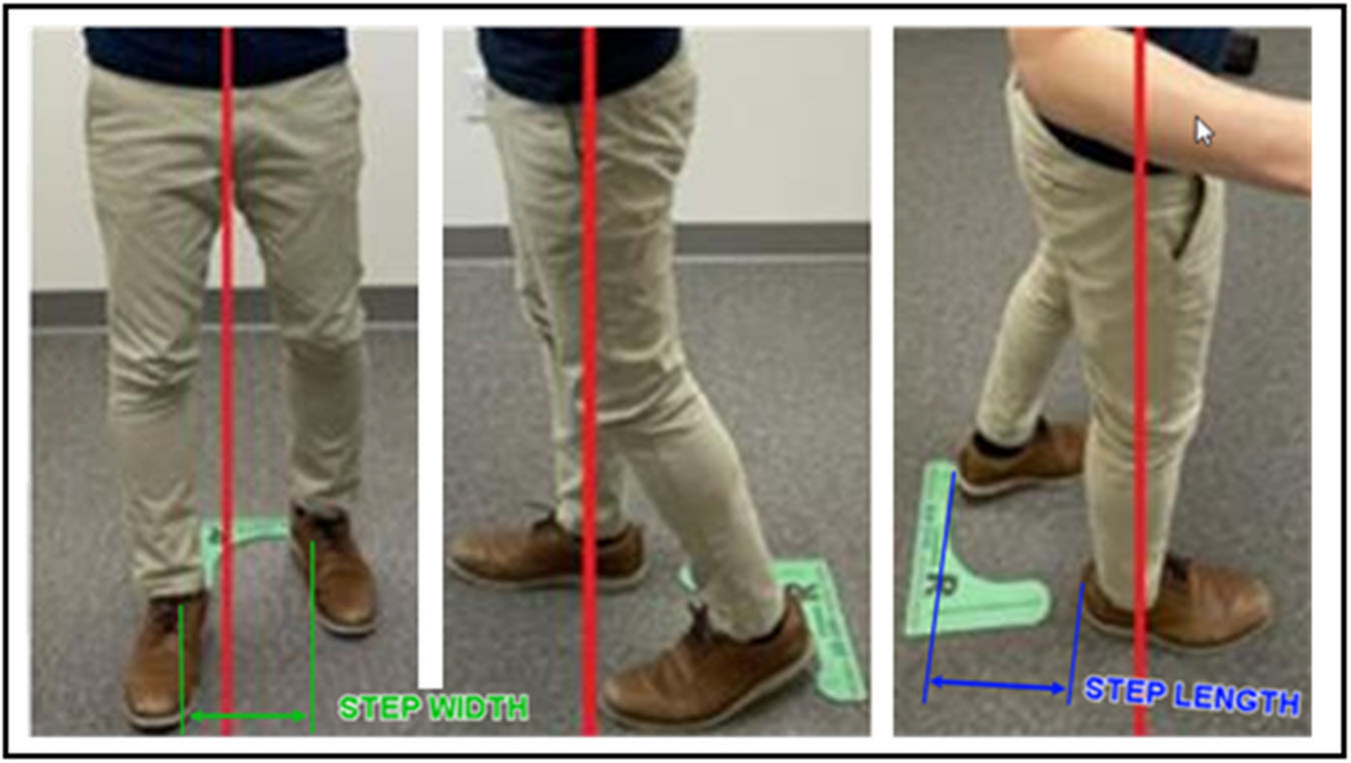
Illustration of step width and length to closely mimic the mid‐stance of the gait cycle.

To mimic the mid‐stance of the gait cycle, the study adopted the methodology done by Abram et al. [2], where eight subjects of body mass: 62.6 ± 9.0 kg; height: 165.3 ± 9.5 cm exhibit the step width of 24 ± 3 cm. Figure 7 displays a step width template designed with a 24 cm reference line. Also, Figure 8 demonstrates the step width and length, where the step width is adjusted within 21–27 cm to closely mimic the mid‐stance of the gait cycle according to the patient's height, weight and comfort at the mid‐stance.

**Figure 9:**
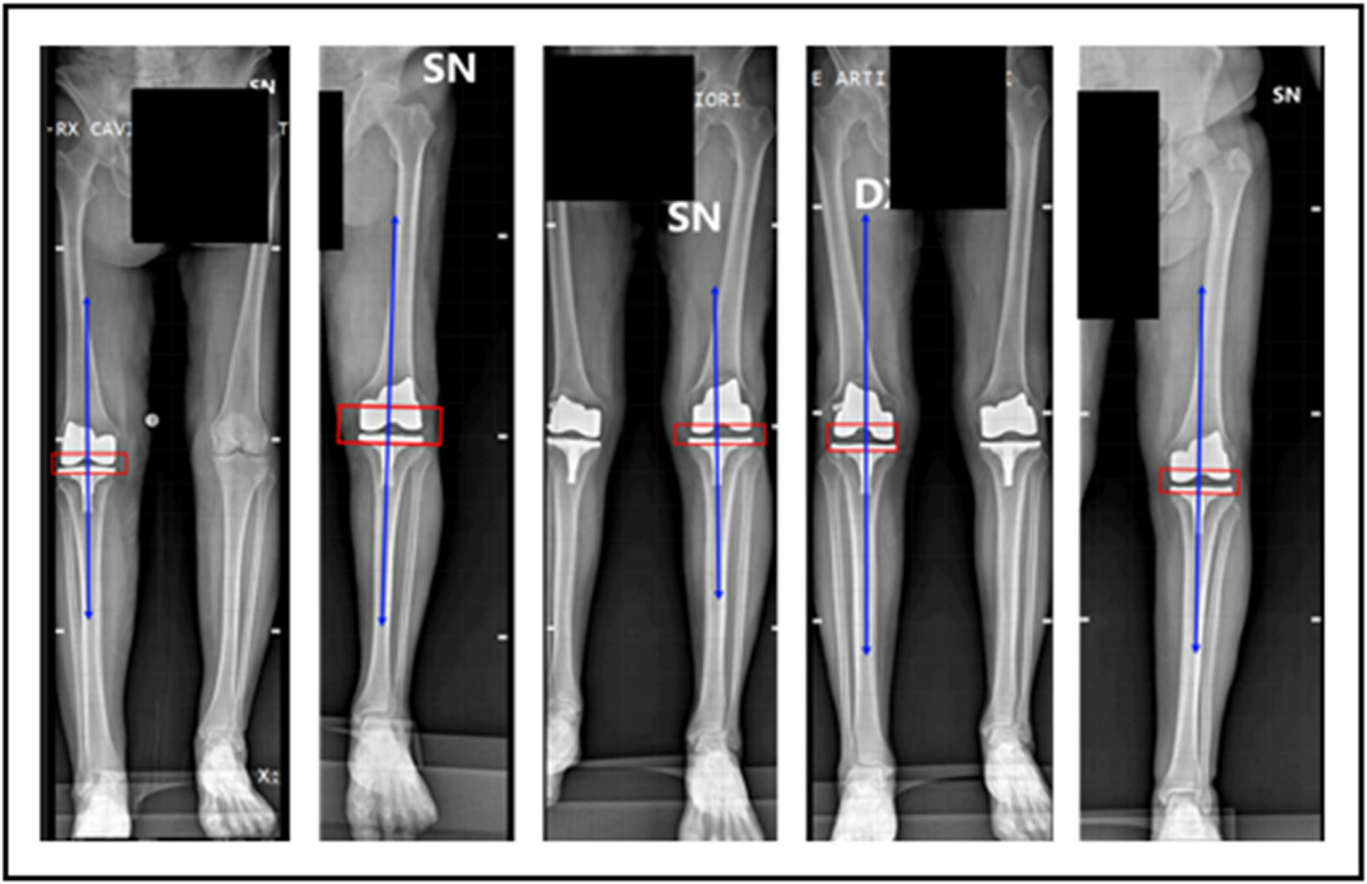
Post‐op radiographs for the validation of physical joint line being parallel to the ground (Symmetric *Smith & Nephew Legion*, Asymmetric *Journey 2*).

Figure 9 displays radiographs analysed to evaluate the alignment of the physical joint line. A total of 12 long leg radiographs were obtained using *GMM Crisis Evolution* radio‐fluoroscopy system. Table 1 shows that the average angle deviation from parallel to the ground is 0.1‐degree varus with the standard deviation of 0.9‐degree. For asymmetric implants, the femoral and tibial resection planes parallel to the physical joint line were used to measure off‐set angles. Due to patient safety considerations, repeated radiographs were avoided. Another factor in the study of the joint line being parallel to the ground is the accuracy of using the *PtoleKnee* cutting guides. Although this study confirms the physical joint line being parallel to the ground, it is estimated that the cutting guides can provide conservatively less than ±1 degree accuracy. These findings confirm the NBA model's reliability and its capacity to achieve alignment parallel to the ground under clinical conditions.

**Table 1:** Joint line angles off from parallel to the ground based on 12 post‐op radiographs.

	Angle off from parallel to ground	Implant
1	0.8° Valgus	Smith & Nephew Journey II
2	1.2° Valgus	Smith & Nephew Journey II
3	0.6° Varus	Smith & Nephew Legion
4	0.6° Varus	Smith & Nephew Journey II
5	0.8° Valgus	Smith & Nephew Journey II
6	0.1° Varus	Smith & Nephew Journey II
7	1.0° Valgus	Smith & Nephew Journey II
8	0.2° Valgus	Smith & Nephew Legion
9	1.7° Varus	Smith & Nephew Journey II
10	0.3° Varus	Smith & Nephew Legion
11	0.2° Varus	Smith & Nephew Journey II
12	1.5° Varus	Smith & Nephew Journey II

**Figure 10:**
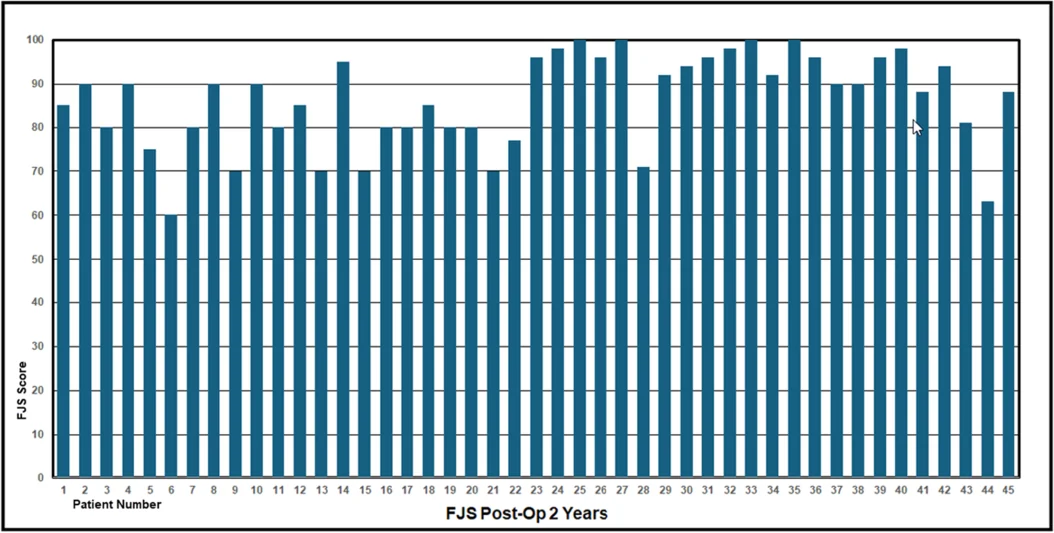
FJS‐12 score of 2‐year postoperative outcome.

Figure 10 exhibits the FJS‐12 of the 2‐year post‐op. The average and standard deviations of 2‐year post‐op are 86 ± 11 (MAX 100, MIN 60). Furthermore, 36 patients have a score greater than or equal to 80 two‐year post‐pp, four patients have measurements of a perfect 100 FJS‐12, and only 2 patients, 6 and 44, have measurements of a FJS‐12 below 70. It should be noted that the sample size of 45 patients is not enough to minimise the influence of chance and allow the authors to draw a meaningful conclusion for the entire TKA population. A sample size of at least 300 would be desirable to draw a more statistically significant conclusion. Therefore, it is recommended to understand the conclusion with caution in this study, and further statistical analysis, such as confidence interval, *p*‐value, and so on, will be studied when the sample size is more than 300 patients for the 2‐year post‐op study. Currently, nearly 300 surgeries have already been performed by four surgeons. It has been reported that none of the surgeries exhibited the laxity and ligament balancing procedure was not required. Furthermore, more long‐leg X‐rays have been obtained and confirmed the concept of NBA, the joint line being parallel to the ground.

## DISCUSSION/CONCLUSION

**Figure 11:**
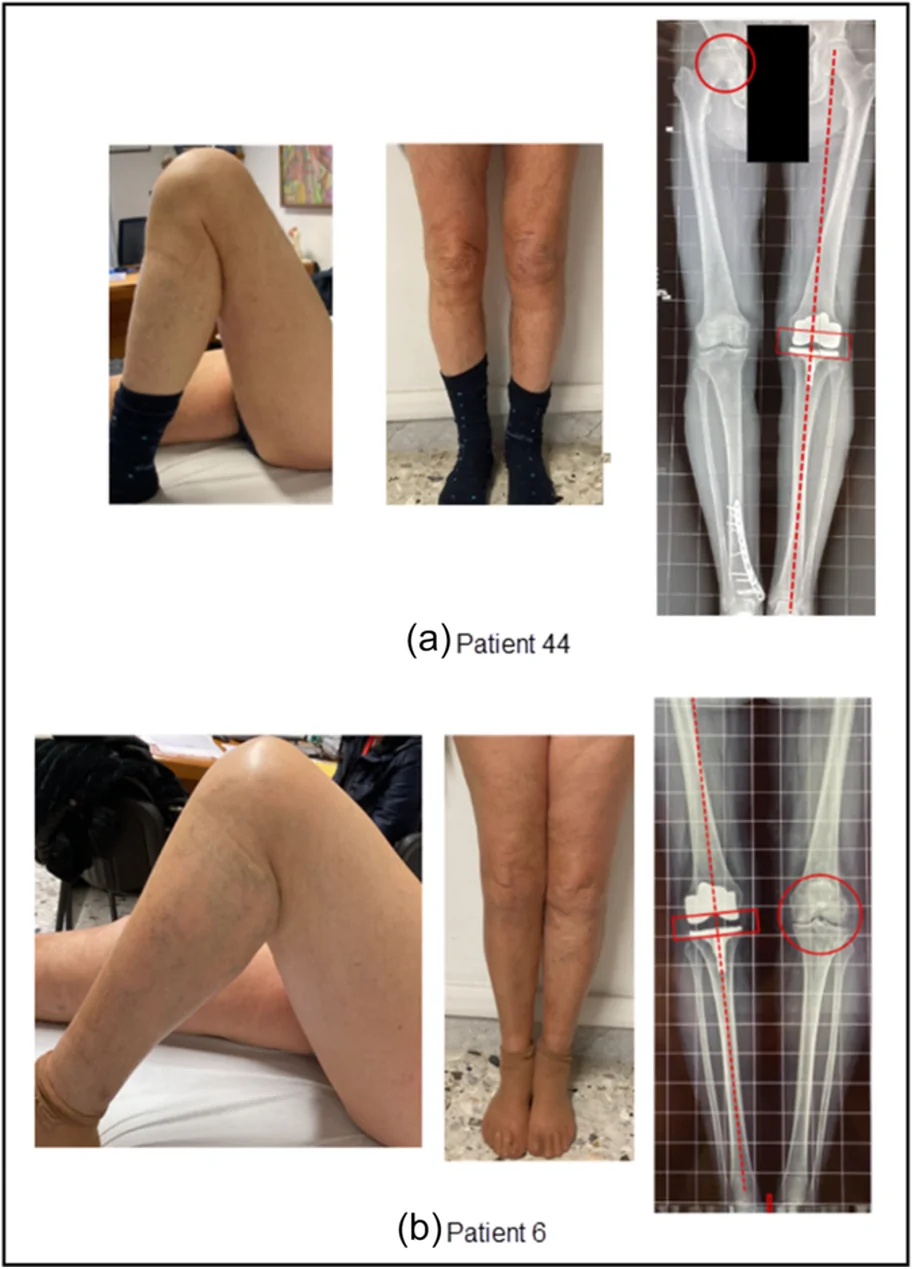
Total knee arthroplasty (TKA) outcome versus potential TKA pain due to the interconnected nature of hip and knee.

In the analysis of FJS‐12, Patient 44 shows the lowest 2‐year post‐op outcome. Patient 44 is a male with a body weight of 77 kg and a height of 1.77 m. As shown in Figure 11a, according to the patient's 6‐month post‐op, the patient's knee flexion is approximately 145 degrees with full extension. However, Figure 11a shows that the hip developed to grade 1 arthritis in his right leg. It now follows that patient 6 is a female with a body weight of 70 kg and a height of 1.6 m. As shown in Figure 11b, according to the patient's 6‐month post‐op, the patient's knee flexion is approximately 125 degrees with full extension. The radiograph, shown in Figure 11b, indicates that her left knee developed to grade 1 or 2 arthritis. The relatively low 2‐year post‐op scores of patients 6 and 44 would be caused by other health issues such as the interconnectedness of the body's musculoskeletal system although it is partly true that TKA would contribute to the patient's perspective on their knee pain and anxiety.

### MA versus NBA

**Figure 12:**
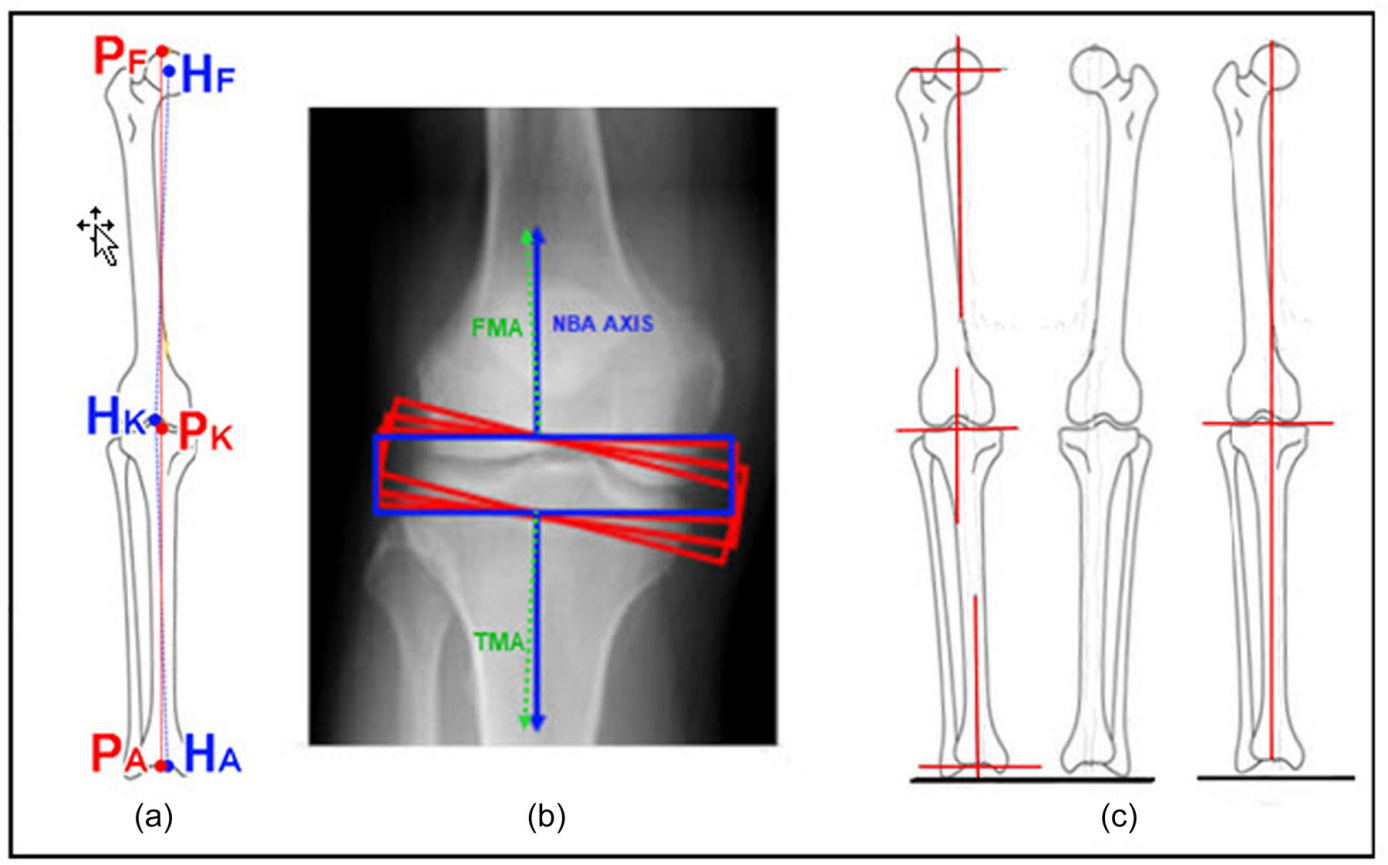
Comparisons of neutral boundary alignment (NBA) to mechanical alignment (MA), kinematic alignment (KA), functional alignment (FA) and anatomical alignment (AA).

NBA is equivalent to MA only when the lower limb is neutral. However, when dealing with outlier lower limbs, MA method is not a suitable method to balance the MCL and LCL of the knee joint, which requires a ligament balancing procedure due to the tissue laxity. As shown in Figure 12a, the offsets of MA femoral head H_F_, knee H_K_ and ankle H_A_ centre with respect to NBA axis can be observed. On the other hand, the NBA axis is based on contact surfaces between the femoral head and the acetabulum P_F_, the distal femur and the proximal tibia P_K,_ and the distal ankle and the proximal talus P_A_. Consequently, if TKA surgeries are performed with the offset information of the centres of the contact surfaces with respect to MA surgical centres, MA would be equivalent to NBA. An ‘outlier’ in TKA refers to an implant or a patient whose anatomy or post‐surgical alignment is significantly different from the norm, which can impact the success of the procedure. It is important to note that a significant difference between a patient anatomical outlier and the norm is based on the definition of femoral and tibial mechanical axes according to MA. However, since the NBA algorithm starts with identifying the characteristics of the lower limb structure, NBA treats all patients as normal as long as they have walked normally prior to the start of osteo‐arthritis symptoms. It is observed that after the lower limb is aligned with NBA, the resection angles of the femur and tibia, with respect to the femoral and tibial mechanical axes, are within 1.3‐degree varus/valgus.

### KA, FA versus NBA

KA and FA methods begin with the femoral and tibial resections close to being parallel to each other (rectangular box formation), promoting MCL and LCL balance in full extension. However, KA typically results in femoral and tibial resections that are slightly varus due to the asymmetric nature of the distal condyles [6]. As a result, the varus positioning of the implant may introduce some shearing effects on the contact surface, which could induce the internal‐external and adduction and abduction muscle activity. Therefore, while MCL and LCL appear to be geometrically balanced, the actual MCL and LCL tensions may not be anatomically balanced, leading to the loss of structural integrity.

FA was recently introduced to align the implant along the mechanical axis to structurally compensate for the varus nature of KA. However, this approach often encounters the same tissue laxity observed in MA (the outlier lower limbs) necessitating ligament balancing procedures. As shown in Figure 12b, if FA and KA resections are rotated and aligned to the NBA axis, FA and KA would become equivalent to NBA.

### AA versus NBA

AA is the alignment method aimed at positioning the femoral and tibial implants parallel to the ground. Although the AA surgical method is well‐described with an emphasis on the joint line being parallel to the ground, it does not explicitly explain the clear reference frame where the joint line is parallel to the ground in a two‐leg stance or one‐leg stance. Furthermore, the conventional definition of the joint line is the line connecting the medial and lateral distal points. It is called the anatomical joint line which is different from the NBA physical joint line. In the case of the two‐leg stance, as shown in Figure 12c, the joint line being parallel to the ground, does not necessarily mean that the implant position is identical to NBA in the direction of gravity. Figure 12c exhibits discontinuity in the direction of gravity at the femoral head, knee and ankle. In other words, although the joint line may be parallel to the ground, the lower limb alignment is not identical to NBA which shows the continuity from the femoral head down to the ankle. Also, the two‐leg stance is not in an extreme position since each leg most likely supports half of the upper body weight. However, a one‐leg mid‐stance is an extreme position where one leg supports the entire upper body weight. Therefore, if AA meets the continuity condition, AA would be equivalent to NBA.

### Static and dynamic equilibrium

**Figure 13:**
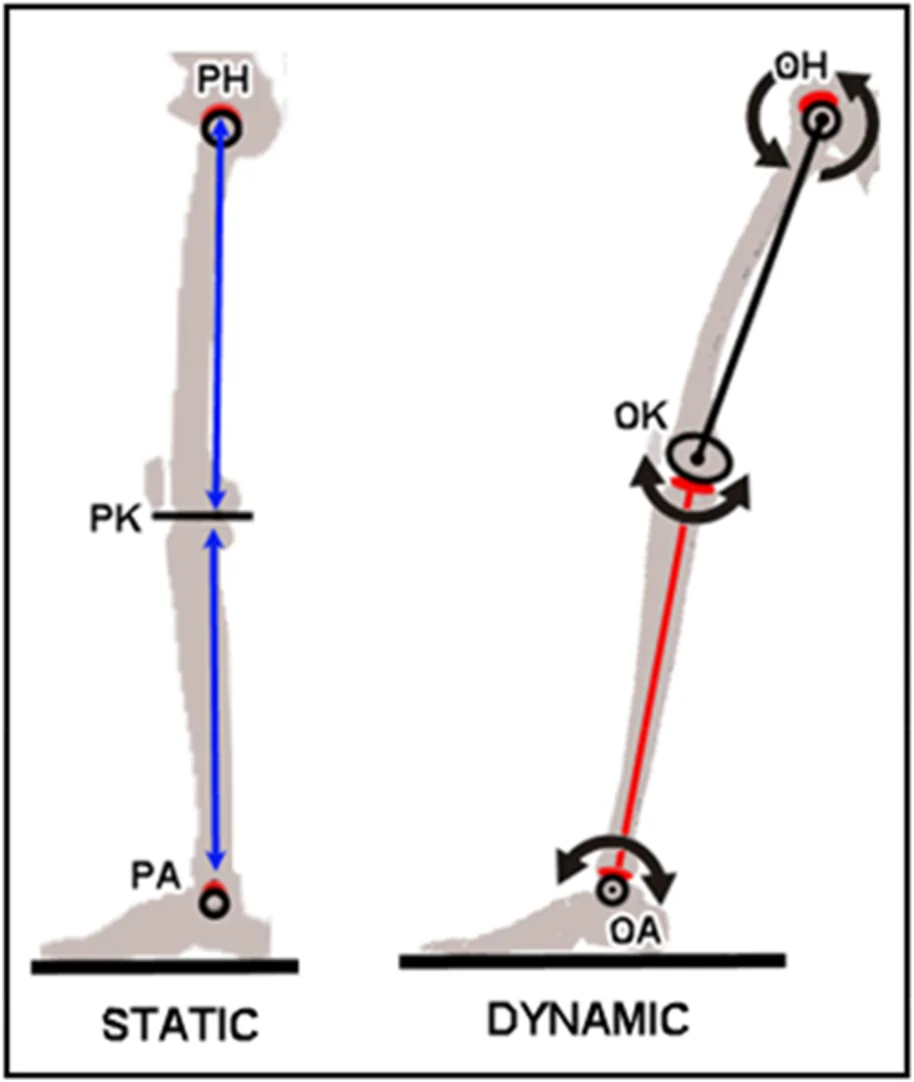
Diagram of static and dynamic equilibriums of the lower limb.

The concept of static and dynamic equilibrium is a core concept and is the heart of the discipline of physics. The study of statics and dynamics is done to determine the force keeping the human body at a stationary position and the instantaneous moment of motion where the sum of the force acting on them is zero in both statics and dynamics. As shown in Figure 13, the motion of the lower limbs starts at the femoral head O_H_, distal femur condyle O_K_ and ankle O_A_ centre. At the quasi‐static position of the mid‐stance, the NBA is analysed based on the contact surfaces of the femoral head to the acetabulum P_H_, the distal femur to the proximal tibia P_K_, and the distal ankle to the proximal talus P_A_. Both analyses of the static and dynamic equilibrium are very important for understanding the lower limb motions for successful TKA surgeries. The first requirement is to position the knee implants along the NBA axis in the direction of gravity. The physical joint line, parallel to the ground, provides not only structural integrity but also knee joint stability and maximum tension of the MCL and LCL in extension of the lower limb at static equilibrium. Consequently, positioning the currently available symmetric and asymmetric implant design is sufficient to provide knee stability at full extension by the femoral and tibial resections perpendicular to the direction of gravity. However, in dynamics, knee joint motion between the femoral and tibial contact surface is quite complex during the flexion involving translation, flexion‐extension, internal‐external and adduction‐abduction rotation [9]. This complex motion is governed by the MCL, LCL, ACL, PCL and patella tendon and the contact surface characteristics of the distal femur to proximal tibia. Therefore, the flexion of the knee joint is a very delicate process out of all the soft tissue and contact characteristics in perfect harmony while satisfying the static equilibrium condition. In other words, the TKA flexion, or dynamic characteristics, strongly depends on the implant contact surface design. If the femoral and tibial implant design closely mimics the patient's knee joint anatomy, the patient can achieve close to his or her own flexion dynamic characteristics. Therefore, it is apparent that the current symmetric or asymmetric implant design could provide excellent flexion results on a group of patients whose contact surface anatomy closely resembles the current available implant design geometry. Otherwise, the post‐op flexion measurement is not as important as the knee joint stability of 0, 45–90‐degree flexion. It should be noted that based on the *Smith and Nephew Journey 2 and Legion* implant design, the clinical data of 250 surgeries demonstrate that the flexion of a minimum 120 degrees would be assured under the NBA. It should be noted that CR implants perform better in patella tracking motion than the PS implants. Furthermore, the asymmetric implant (*Smith and Nephew Journey 2*) shows no significant difference compared to the symmetric implant (*Smith and Nephew Legion*) in terms of implant stability and surgical outcome.

In this study, 3‐D geometric modelling of the lower limb is presented. The physical joint line, parallel to the ground and perpendicular to the NBA axis in the direction of gravity, is defined at the mid‐stance of the gait cycle where a one leg stance supports the entire weight of the upper body and the MCL and LCL reach their full balanced tension to provide full extension knee stability. The clinical radiographs highlight the clinical data which demonstrates consistent TKA outcomes from NBA despite the small sample size (*N* = 45). NBA theory provides information regarding implant positioning at static equilibrium. However, further research should be continued to introduce a better and more comprehensive 3D model of the knee joint and knee implant design accommodating the lower limb dynamics. Furthermore, to draw a more statistically significant conclusion, a 200 2‐year post‐op analysis is being conducted and will be confidently published within 2 years.

## AUTHOR CONTRIBUTIONS

Bruno Violante, Lorenzo Deveza and Ilwhan Park contributed to the development of the theory. Bruno Violante, Francesco Pollara, Giovanni Rusconi, Gianroberto Ferreri and Alessandro Annibaldi contributed to clinical validation, Lorenzo Deveza, Francesco Pollara and Ilwhan Park contributed to writing the manuscript. Bruno Violante, Alessandro Annibaldi, Giovanni Rusconi and Gianroberto Ferreri read and approved the manuscript. All the authors read and approved the final manuscript.

## CONFLICTS OF INTEREST STATEMENT

Bruno Violante royalties and consulting agreements with Smith and Nephew, Stock options with Lento Medical, Inc. Lorenzo Deveza has consulting and stock option interests with Lento Medical, Inc, Ownership interests with IMNovations, Inc, and Lunas Ilwhan Park is employed with Lento Medical, Inc. The remaining authors declare no conflicts of interest.

## ETHICS STATEMENT

This study was approved under Baylor Institutional Review Board #h‐55527.

## Data Availability

The data are available upon reasonable request to the corresponding author.
